# Antimicrobial effect of curcumin, alone or in combination with black pepper, against foodborne pathogens in vacuum-packed ground mutton

**DOI:** 10.1038/s41598-025-08350-2

**Published:** 2025-07-08

**Authors:** Mohamed Anwar Elghareeb, Hend Ali Elshebrawy, Hanan Ahmed Zaher, Khalid Ibrahim Sallam

**Affiliations:** Department of Food Hygiene, Safety, and Technology, Faculty of Veterinary Medicine, Mansoura University, Mansoura, 35516 Egypt

**Keywords:** Mutton, Vacuum packaging, Curcumin, Black pepper, Foodborne pathogens, Shelf life, Sensory attributes, Biotechnology, Microbiology

## Abstract

Finding healthy, effective, safe, and affordable natural alternatives to synthetic preservatives for extending the shelf life of meat products has become a research priority. The present study aimed to investigate the effects of curcumin (0.3%, 0.7%, and 1%), black pepper (0.3%), or their combinations on the sensory attributes, shelf life, spoilage bacterial counts, and foodborne pathogens in vacuum-packed ground mutton stored at 4 °C. Ground mutton treated with a mixture of black pepper and curcumin maintained their quality and acceptability for up to 21 days compared to only 7 days for the controls. By day 21 of storage, ground mutton treated with the combination of 0.3% black pepper and 1% curcumin exhibited significant reductions in anaerobic, lactic acid, and psychrotrophic bacterial counts by 2.37, 2.05, and 2.3 logs, respectively, along with significant reductions in the inoculated *Salmonella* Typhimurium and *Escherichia coli* O157:H7 by 2.75 and 2.39 logs, respectively compared to the control. Interestingly, a complete inhibition of the inoculated *S*. *aureus* (MRSA) growth was observed in ground mutton treated with 0.3% black pepper and 1% curcumin blend by the 14th day of storage. Thus, curcumin and black pepper may serve as effective natural preservatives for prolonging shelf life and controlling foodborne pathogens in meat products during vacuum-packaged storage.

**Supplementary Information:**

The online version contains supplementary material available at 10.1038/s41598-025-08350-2.

## Introduction

Sheep meat is a leading source of high-quality protein (about 20 g/ 100 g lean meat), heme–iron, zinc, selenium, and B complex vitamins, especially highly bioavailable vitamin B12^1^. Around 100 g of sheep meat contains 100–200% of the recommended daily intake of vitamin B12, 40% of vitamin B3, and 10–20% of iron, zinc, selenium, and vitamin B6^2^. Mutton is the meat derived from an adult, mature sheep over one year old. Mutton is the second most popular red meat consumed in Egypt after beef. Globally, many countries, including Turkmenistan, Iceland, New Zealand, Mauritania, Arab Gulf states, and Australia, consume high amounts of mutton. Mongolia is the top country in sheep meat consumption, with an estimated amount of 49.8 kg/capita/year in 2023 (https://www.reportlinker.com/dataset/51beb21fae2c8687633cf347eb2175777295c816). Most people in many Arabian and African countries prefer sheep meat over other meat types due to its delicious flavor, tender texture, unique organoleptic characteristics, and nutritive value.

Meat is a nutritious food with high water content and is susceptible to microbial spoilage even when refrigerated. The muscle from healthy sheep is nearly sterile, but its meat becomes contaminated with spoilage bacteria and foodborne pathogens during various handling procedures, starting with slaughtering and evisceration procedures and ending with the transportation and storage of meat^3^. Microbial spoilage deteriorates the meat quality by causing slime formation, off flavors, and discoloration, rendering it unfit for human consumption. Among the psychotropic bacteria that grow on meat at cold temperatures are gram-negative bacteria, such as *Pseudomonas* spp. and *Enterobacteriaceae*, as well as Gram-positive ones, like lactic acid bacteria. Moreover, lactic acid bacteria (LAB), including *Lactobacillus* and *Leuconostoc*, can significantly contribute to the spoilage of chilled raw meat and can be a leading competitor of other types of spoilage bacteria^4,5^.

Foodborne pathogens, such as *Salmonella* spp., *Escherichia coli* O157:H7, and *Staphylococcus aureus*, are major global causes of illness and mortality, presenting significant public health and economic challenges. Among these, *Salmonella* is the leading cause of foodborne diseases, accounting for 1.35 million infections, 26,500 hospitalizations, and 420 deaths annually in the USA^6^. Similarly, *E*. *coli* O157:H7 and *S*. *aureus* are responsible for approximately 3.6% and 2.6% of foodborne illnesses annually in the United States, representing a severe economic loss^7^. Conversely, there is no available national statistical data regarding the socioeconomic impact of foodborne pathogens on healthcare systems in Egypt. Mutton meat may become contaminated with these foodborne pathogens during various processing stages, posing a serious public health risk^8^. Therefore, there is an urgent need for effective, safe, and natural preservatives to combat these pathogens and prevent foodborne illnesses.

Various methods have been implemented in the food industry to extend shelf life, enhance sensory qualities, and prevent the growth of spoilage bacteria and foodborne illnesses. Vacuum packing and natural preservatives have gained considerable attention due to their effectiveness and ease of use. Conventional vacuum packaging (VP) creates anaerobic conditions, hindering the development of aerobic microorganisms and retarding the growth of facultative ones, thus extending the shelf life of meat and meat products^9^. Furthermore, VP has proven more effective than aerobic packaging in controlling microbial spoilage in marinated and unmarinated camel meat^10^.

Over the years, synthetic chemical additives have been used to preserve meat, posing potential health hazards due to their carcinogenic and toxic properties. Consequently, using natural food additives, such as herbs (onion, garlic, turmeric, thyme, rosemary, coriander, and oregano), or essential oils presents a healthier choice, which gained wide acceptance among consumers as they can inhibit the growth of foodborne pathogens and enhance the sensory attributes of food without any negative consequences on human health^11^.

Curcumin is a yellow polyphenolic pigment and the main active compound in turmeric, derived from the Curcuma longa rhizome, a member of the ginger family^12^. It comprises about 3.14% of powdered turmeric, giving it its vibrant yellow color^13^. Curcumin, also known as CI 75,300, Natural Yellow 3, or diferuloylmethane, is recognized as a food additive with the code E100 that is commonly used for its coloring, flavoring, and preserving properties^14^. Turmeric products are considered safe by the FDA and FAO/WHO^15^. Curcumin is believed to have broad-spectrum antimicrobial activity against Gram-positive and Gram-negative bacteria, including foodborne pathogens, by inhibiting the pathogen’s virulence factors and increasing host-mediated immunity^12,16^. However, its oral bioavailability is low due to poor intestinal absorption and rapid degradation^17^. Adding compounds like piperine (The main bioactive constituent in black pepper) can significantly increase curcumin’s bioavailability by up to 2000%^18^.

Black pepper (*Piper nigrum* L.), often referred to as “The King of Spices,” is the most widely used spice globally and belongs to the *Piperaceae* family. The pungency and aroma of black pepper are mainly related to alkaloid piperine, volatile oil, and oleoresins^19^. Black pepper is a rich source of essential vitamins, minerals, and nutrients. For example, 100 g of black pepper seeds provide 10 g of protein, 66.5 g of carbohydrates, 10.2 g of fat, and substantial amounts of calcium, magnesium, potassium, iron, and zinc. It also contains notable concentrations of vitamins C, B1, B2, and B3^20^. Black pepper extracts can efficiently inhibit the growth of Gram-positive and Gram-negative bacteria, including *S*. *aureus*, *Vibrio cholerae*, *Bacillus cereus*, *Shigella dysenteriae*, *Streptomyces faecalis*, *Escherichia coli*, *S*. Typhimurium, *Listeria monocytogenes*, and *Pseudomonas* spp.^21,22^.

To date, there is scarce data about using curcumin as a natural food additive for inhibiting the food spoilage bacterial growth and foodborne pathogens in food. Only two publications are available concerning the antimicrobial effect of curcumin in meat; the first one concerned its inhibitory impact on *Salmonella* in chicken meat^23^, and the other publication regarded its inhibitory effect on *L*. *monocytogenes*, *S*. *aureus*, *S*. *Typhimurium*, and *E*. *coli* O157:H7 pathogens in veal minced meat^24^. No studies are available concerning the antimicrobial effect of curcumin, either alone or in combination with black pepper, against naturally occurring food spoilage bacteria, as well as the artificially inoculated foodborne pathogens in vacuum-packed ground mutton under refrigerated storage.

This study is the first aimed to determine the efficacy of using different concentrations of curcumin (0.3%, 0.7%, and 1%) and black pepper (0.3%) to inhibit the growth of spoilage bacteria (anaerobic bacteria, lactic acid bacteria, and psychrotrophic bacteria) and foodborne pathogens (*Salmonella enterica* serovar Typhimurium, *Escherichia coli* O157:H7, and *Staphylococcus aureus*); as well as their ability to enhance the sensory attributes and extend the shelf life of vacuum-packed ground mutton stored at 4 °C for 21 days. The present research also intended to investigate black pepper’s ability to improve the sensory attributes scores and antimicrobial effects of curcumin by combining three different concentrations of curcumin (0.3%, 0.7%, and 1%) with a constant ratio of black pepper (0.3%).

## Materials and methods

### Samples collection and preparation

The experiment of sensory evaluation and microbiological examination of ground mutton was done on three independent occasions at different times, during which triplicate samples from each of the control and treated mutton samples were tested. On each occasion, twenty-four kilograms of freshly ground mutton were purchased from a reputable butcher shop in Mansoura City, Egypt. The collected samples were carefully packaged into sterile impermeable polyethylene bags and promptly transported in an isothermal box to the laboratory of the Food Hygiene, Safety, and Technology Department, Faculty of Veterinary Medicine, Mansoura University, Egypt, where the antimicrobial effects of curcumin and black pepper against spoilage bacteria and foodborne pathogens were examined.

Curcumin and black pepper powder were purchased from a reliable herbal store in Mansoura City, Egypt. The curcumin powder was labeled as 100% organic and pesticide-residue free, containing approximately 95% total curcuminoids. The ground mutton was divided into eight groups (a control group and 7 treatment groups), each weighing three kilograms. The eight groups included a control without curcumin or black pepper and seven treated groups comprising 0.3% curcumin powder, 0.7% curcumin powder, 1% curcumin powder, and 0.3% black pepper, along with mixtures of 0.3% black pepper and 0.3% curcumin powder, 0.3% black pepper and 0.7% curcumin powder, and 0.3% black pepper and 1% curcumin powder. The concentrations of curcumin and black pepper were determined through preliminary trials to assess sensory acceptance in cooked ground mutton.

**Fig. 1:**
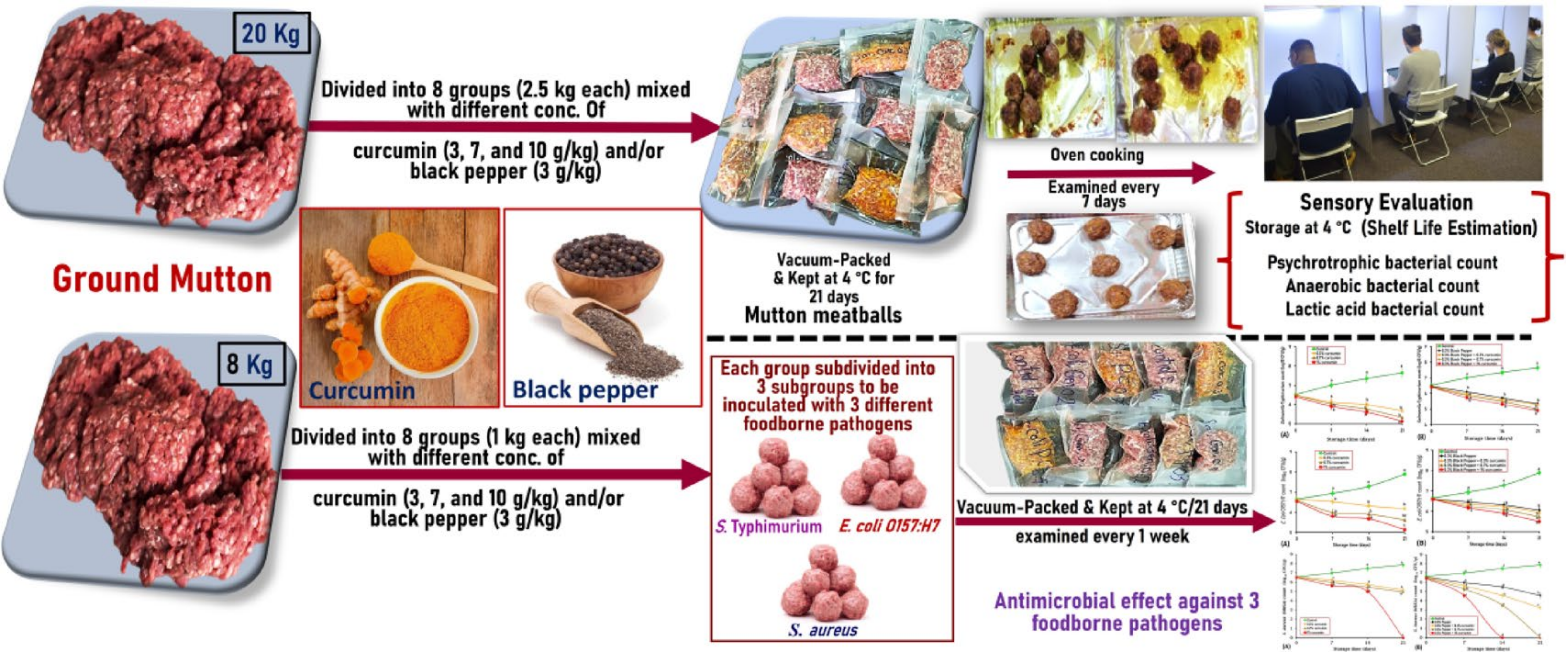
A graphical abstract of the study design, workflow, and results.

Curcumin and black pepper were added to each ground mutton group and mixed thoroughly by hand with gloves for three minutes until a homogeneous mixture formed. Subsequently, the treated meat was shaped into meatballs weighing approximately 30 g each. The prepared mutton meatballs were packaged in a food vacuum packaging pouch made of polyamide plastic and vacuum-sealed using an automatic vacuum machine. The vacuum-packed mutton meatballs, including the control and seven treated groups, were stored at 4 °C for 21 days to evaluate their sensory attributes, shelf life, and microbiological quality, including anaerobic plate count, lactic acid bacteria count, and psychrotrophic bacterial count. An overview of the study design is shown in Fig. 1.

All methods were carried out in accordance with the guidelines and regulations of the Mansoura University-Research Ethics Committee, which are consistent with the WMA Declaration of Helsinki—Ethical Principles for Medical Research Involving Human Participants.

All experimental protocols were approved by the Mansoura University Animal Care and Use Committee (MU-ACUC) under the proposal code of “VM.MS.22.09.3”.

### Sensory evaluation

Sensory evaluation was carried out for the control mutton meatball samples and the treated samples with curcumin, black pepper, or curcumin-black pepper combination (7 treatment lots) at seven-day intervals throughout a 21-day storage period. Mutton meatballs (30 g each) from each group were cooked in clean aluminum foil plates at 180 °C for 20 min using an electric cooking oven. The cooked samples were coded with randomly selected 3-digit numbers and presented to a panel of twenty-one semi-trained panelists (16 females and 5 males, aged between 22 and 45 years) comprising members of the Food Safety, Hygiene, and Technology Department, Faculty of Veterinary Medicine, Mansoura University. Panelists were trained in the sensory profiling of mutton over 5 training sessions. Before participation, all participants provided consent by checking the option, “I understand my responses are confidential and agree to participate.” Participants can withdraw from the investigation at any time without explanation. All examined meat samples were confirmed safe for consumption. A nine-point hedonic scale ranging from 1 (extremely disliked sample) to 9 (extremely liked sample) was employed to assess the flavor intensity, tenderness, juiciness, and overall acceptability of the control and treated meatball samples^25^.

### Microbiological examination

#### Sample preparation for microbiological examinations

On days 0, 7, 14, and 21 of storage, 10 g from the control and the seven treated ground mutton groups were taken and blended with 90 ml of sterile 0.1% peptone water (Oxoid CM0009) in a laboratory blender for 1 min. Decimal serial dilutions up to 10^−6^ were prepared using the method recommended by ISO (2017)^26^.

#### Anaerobic plate count, lactic acid bacteria count, and psychrotrophic bacterial count for shelf life determination

The anaerobic plate count (ANPC) for both control and treated ground mutton samples was enumerated by spreading 0.1 ml of the appropriate dilutions onto the surface of plate count agar medium (CM0325, Oxoid, Hampshire, UK) and incubating under anaerobic conditions (with gas-producing kits in an anaerobic jar) at 30 °C for 3 days^27^. For lactic acid bacteria (LABC), 0.1 ml from the appropriate dilution was plated onto the surfaces of de Man, Rogosa, and Sharpe Agar plates (MRS Agar, Himedia M641, India) and incubated under anaerobic conditions for 72 h at 30°C^28^. The psychrotrophic count (PPC) was determined after spreading 0.1 ml of the previously prepared meat dilutions on duplicate plate count agar plates (CM0325, Oxoid, Hampshire, UK) and incubating for 7 days at 5°C^29^. Bacterial colonies were then enumerated and counted as log_10_ CFU/g.

#### Antimicrobial effect of curcumin and black pepper on foodborne pathogens

##### Bacterial strains and inoculum preparation

Three foodborne pathogens isolated previously from meat products in our laboratory were used in the current study, including *Salmonella enterica* serovar Typhimurium, *Escherichia coli* O157:H7, and *Staphylococcus aureus*. All bacterial strains used in the study exhibited high resistance to antimicrobials and had a Multiple Antibiotic Resistance (MAR) index of ≥ 0.56. *Salmonella* Typhimurium and *E. coli* O157:H7 were resistant to β-lactams, quinolones, and sulfonamides, while *Staphylococcus aureus* exhibited resistance to macrolide-lincosamide-streptogramin B (MLSB) and fluoroquinolones.

The isolation and identification of the three selected foodborne pathogens were carried out according to the International Organization for Standardization recommended techniques for *S*. Typhimurium^30^, *E. coli* O157:H7^31^, and *S*. *aureus*^32^. Each strain used was revived on tryptone soy broth (Oxoid CM0129) and incubated at 37 °C for 18–36 h. The inoculation level of the bacterial suspension for *S*. Typhimurium, *E*. *coli* O157:H7, and *S*. *aureus* was calculated after counting on the corresponding selective media, which was found to be 9.86 ± 0.37, 10. 26 ± 0.45, and 10.15 ± 0.42 log_10_ CFU/ml, respectively.

##### Inoculation of curcumin and black pepper-treated ground mutton with foodborne pathogens

An additional eight kilograms of ground mutton were purchased from the same butcher shop and immediately transferred to the laboratory of the Food Hygiene, Safety, and Technology Department, Faculty of Veterinary Medicine, Mansoura University, Egypt, to study the effect of curcumin, black pepper, or their combination on the growth of *S*. Typhimurium, *E*. *coli* O157:H7, and *Staphylococcus aureus* (methicillin-resistant *S*. *aureus*; MRSA). The procured ground mutton (8 kg) was divided into 8 groups (one kilogram each), encompassing a control group without curcumin and black pepper and 7 treatment groups. Each of the eight groups was further divided into three subgroups (about 330 g each) and inoculated with either *S.* Typhimurium, *E. coli* O157:H7, or *S. aureus*. Each meat subgroup (330 g each) was inoculated with 3.3 ml (approximately 10 log_10_ CFU/mL) from one of the three previously prepared foodborne culture pathogen inoculum containing *Salmonella enterica* serovar Typhimurium, *E. coli* O157:H7, or *S*. *aureus*. Pathogen-inoculated meat samples were thoroughly mixed by hand using sterile gloves for two minutes to achieve a homogeneous paste containing nearly 8 log_10_ CFU/g of the foodborne pathogen. The current study used a final contamination level of approximately 8 log_10_ CFU/g to accurately quantify the range of log reductions from used treatments during storage.

The artificially inoculated ground mutton samples were shaped into meatballs weighing 30 g each and packaged in a food vacuum packaging pouch. The pathogen-inoculated mutton meatballs, including the control and seven treatment groups, were vacuum-sealed and stored at 4 °C for 21 days to be evaluated every seven days during the storage period by blending 10 g of meatball samples with 90 mL of sterile peptone water (0.1%) for 45 s to prepare tenfold serial dilutions and recovery of inoculated pathogens was done using the standard spread plate technique in their corresponding selective media. The selective agars used were Xylose Lysine Deoxycholate (XLD Agar, CM0469, Oxoid Ltd.), Sorbitol MacConkey Agar supplemented with cefixime and potassium tellurite (Oxoid, CM0813, SR0172), and Baird-Parker selective agar with egg-yolk tellurite emulsion (Oxoid CM275, S00R54) for the recovery and enumeration of *S*. *enterica* serovar Typhimurium, *E*. *coli* O157:H7, and *S*. *aureus*, respectively. All inoculated plates were incubated at 37 °C for 24 h. The characteristic colonies of the three different bacterial pathogens were enumerated, and the counts were expressed as log_10_ CFU/g of meat.

### Statistical analysis

Experiments were conducted on three independent occasions over 4 months. Statistical analysis was performed using SPSS Statistics Version 21.0 (SPSS Inc., Chicago, IL, USA), and results reported as mean ± standard error of three replicates. One-way ANOVA was used to assess significant differences in microbial counts across various treatments. The General Linear Model (GLM) was employed to analyze the sensory attribute scores. Tukey’s HSD (honestly significant difference) test was applied to compare the statistical significance of mean differences among the treatments. Differences between the mean values were considered significant at *P* < 0.05 or *P* < 0.01.

## Results and discussion

### Sensory evaluation

**Fig. 2:**
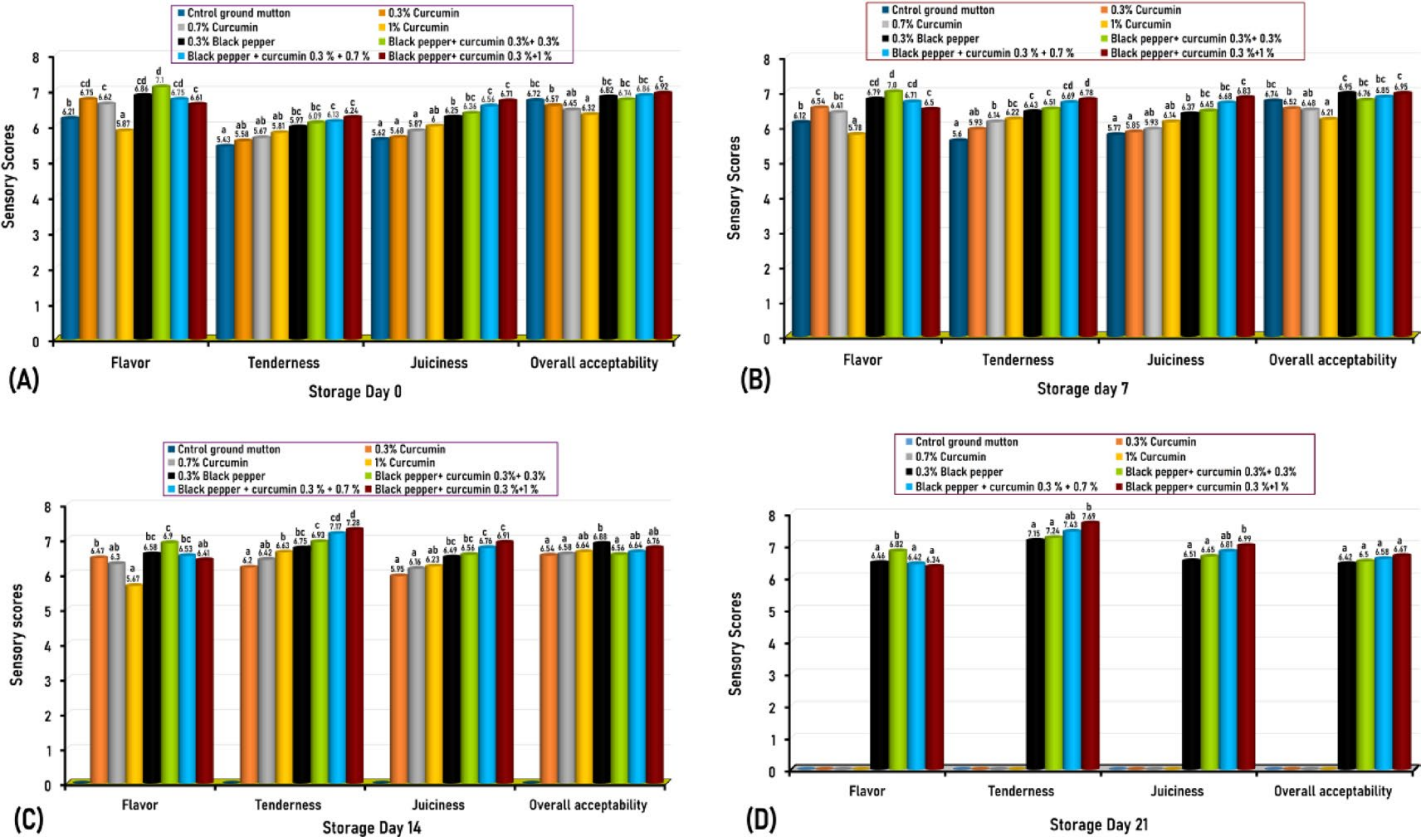
Mean values of the sensory characteristic scores of cooked mutton meatballs treated with different concentrations of curcumin either alone or in combination with black pepper during vacuum-packed storage at 4 °C for 21 days. Mean values of 3 independent occasions given by 21 semitrained panelists during day 0 (**A**), day 7 (**B**), day 14 (**C**), and day 21 (**D**). Mean values with different letters among the various treated groups in each studied attribute are significantly different (*P* < 0.05). A nine-point hedonic scale from 1, which stands for extremely dislike, to 9, which stands for extremely like sample was used to determine the flavor, tenderness, juiciness, and overall acceptability.

Sensory attributes of oven-cooked mutton meatballs treated with different concentrations of curcumin, black pepper, and curcumin-black pepper blend are shown in Fig. 2. Sensory evaluation is a prime index for determining the shelf life of food, as a decrease in sensory quality influences food acceptability and can render it inedible to consumers. A panel of 21 semi-trained panelists participated in the sensory evaluation. Panelists were trained on the sensory profiling of mutton products, focusing on color, odor, taste, texture, and overall acceptability using a 9-point hedonic scale (1 = dislike extremely, 9 = like extremely). Sensory evaluations were conducted on storage days 0, 7, 14, and 21. Sensory attributes were not assessed from day 14 for control samples (without any treatment) and on day 21 for mutton meatballs treated with 0.3%, 0.7%, and 1% curcumin due to the noticeable deterioration in color and odor (Fig. 2).

Samples were deemed unacceptable when the mean sensory scores for odor or overall acceptability fell below 4 (dislike slightly) on a nine-point hedonic scale. The sensory attributes of the control sample were not assessed from day 14 due to off-odor, slime formation, and off-colors, which are closely linked to microbial spoilage indicators, particularly lactic acid bacteria, that reached 7.02 log_10_ CFU/g on day 14 of storage. Similarly, Ercolini et al.^4^ noted that microbial loads of 7 log_10_ CFU/g are typically associated with off-odors and off-flavors, with lactic acid bacteria playing a significant role in the spoilage of refrigerated raw meat. Conversely, curcumin-treated mutton samples were not sensory evaluated on day 21 due to deteriorative changes in color and odor, even though their microbial load had not reached 7 log_10_ CFU/g. In this regard, Borch et al.^33^ indicated that the correlation between bacterial counts, particularly lactic acid bacteria, and sensory spoilage is not always precise, complicating the use of bacterial levels as indicators of spoilage.

The present study demonstrated that the sensory attributes of the vacuum-packed control sample, stored at 4 °C remained acceptable without any deteriorative changes until the 7th day of the experiment (Fig. 2), which aligns with the previous findings of Gertzou et al.^34^, who reported a shelf life of 10 days for fresh chicken legs, packed in polyamide/polyethylene bags under vacuum sealing during storage at 4 °C. Furthermore, using vacuum packaging as a preservation method minimizes the exposure of meat products to oxygen, consequently slowing down oxidative reactions and microbial growth, which are primary factors contributing to spoilage. This method helps maintain the quality and safety of the meat by preserving its sensory characteristics, such as color, odor, and texture, for a longer duration compared to conventional packaging^9,10^.

Generally, the present study showed that incorporating curcumin (a bioactive compound found in turmeric), black pepper, or their combination into mutton meatballs effectively prolonged the meat’s shelf life during vacuum-packed storage at 4 °C compared to the control sample (Fig. 2). In this context, Bae et al.^35^ revealed that 0.5% turmeric powder improved the storage condition of pyeonyuk (a traditional Korean meat dish) compared to the control sample (without any treatment) by inhibiting lipid oxidation and suppressing microbial growth. Furthermore, Pezeshk et al.^36^ found that rainbow trout samples treated with 3.5% turmeric extracts retained good quality until the 15th day of storage and remained acceptable until day 20 versus day 15 for the control samples (without turmeric), which exhibited spoilage characterized by off-odor, slime formation, and discoloration due to high microbial growth and lipid oxidation. On the other hand, black pepper is widely known for extending the shelf life of meat through its antimicrobial properties and preserving meat quality by reducing lipid oxidation via its antioxidant effects^21,22,37^. Curcumin and black pepper, whether used separately or together, could extend the meat’s shelf life by enhancing their antioxidant and antimicrobial properties^38^.

Throughout the experiment, ground mutton treated with 1% curcumin exhibited a significant decrease (*P* < 0.05) in flavor acceptability compared to other samples. Meanwhile, a mixture of 0.3% black pepper and 0.3% curcumin resulted in an obvious improvement in flavor acceptability compared to the other samples tested (Fig. 2). There was no significant difference (*P* > 0.05) observed in the overall acceptability between the control and treated mutton meatballs, except for 1% curcumin-treated samples that showed the lowest score and the 0.3% curcumin and 1% curcumin that revealed the highest score, during days 0 and 7 of storage. It is noticeable that treated mutton meatballs with black pepper alone or in combination with curcumin were more acceptable than meatballs treated with curcumin alone (Fig. 2). On day 14 of storage, there was a significant difference in overall acceptability between the 0.3% curcumin-treated sample, which showed the lowest score, and each of the samples treated with 0.3% black pepper and the blend of 0.3% black pepper and 1% curcumin, which exhibited the highest score. On day 21 of storage, samples tested (treated with black pepper alone or in combination with curcumin) showed no significant difference in their overall acceptability (Fig. 2). Curcumin, derived from *Curcuma longa L*., is characterized by its vibrant yellow-orange color, slightly sharp odor, and bitter taste. It is used in the food industry (E100) as a preservative, colorant, and flavor enhancer^14^. The addition of 1% curcumin to mutton meatballs resulted in a slightly lower acceptability score compared to the other treated samples, owing to its slight bitterness and faint, sharp odor, which was observed by panelists in terms of flavor, reflected in flavor scores ranging from 5.67 to 5.87 (meaning; neither like nor dislike) on the nine-point hedonic scale during the storage period. However, 1% curcumin is insufficient to alter the meat color. Black pepper is widely used worldwide due to its appealing taste, enhancing the overall acceptability of meat. Combining curcumin and black pepper can mitigate curcumin’s bitterness and impart unique flavors and aromas, improving the sensory profile of meat (Fig. 2). Similarly, chicken meat treated with 1% and 2% curcumin was accepted by the panelists for its flavor and appearance; however, the 3% curcumin treatment resulted in a too-yellow color and a sharp, bitter taste, rendering it unacceptable according to the panelists’ evaluation^23^. Conversely, Bae et al. found that adding 0.5% turmeric powder to pyeonyuk showed no significant differences in flavor and total acceptability of treated samples^35^.

There was no significant difference in tenderness or juiciness between control and curcumin-treated mutton meatballs; nevertheless, curcumin-treated meatballs showed a slight improvement in both tenderness and juiciness compared to the control sample (Fig. 2). In contrast, grilled mutton meatballs treated with curcumin-black pepper mixture (0.3% black pepper + 0.3% curcumin, 0.3% black pepper + 0.7% curcumin, and 0.3% black pepper + 1% curcumin) revealed significant (*P* < 0.05) improvement in tenderness and juiciness compared to the control and other treated samples, with the blend of 0.3% black pepper + 1% curcumin demonstrated the highest scores in tenderness and juiciness (Fig. 2). The improvement in tenderness and juiciness of treated samples of the black pepper-curcumin blend may be linked to the ability of the two spices to retain moisture, contributing to improved juiciness and tenderness. Furthermore, Gulel et al. found no significant difference in the texture of chicken meat samples treated with 1%, 2%, and 3% curcumin^23^. In this context, Ashayerizadeh et al. revealed that adding black pepper and turmeric powder to quail diets enhances carcass characteristics and meat quality and found that a combination of 0.5% turmeric powder and 0.5% black pepper effectively controls oxidation reactions, maintains water storage between myofibrils, and increases muscle water-holding capacity, improving meat taste and tenderness^38^. Consequently, curcumin, black pepper, and their combination can be used as promising natural additives for meat preservation and flavor enhancers under refrigerated storage.

### Antimicrobial effect of curcumin and black pepper against spoilage bacteria

Vacuum packing and natural preservatives, especially under refrigerated storage, are widely used to extend the shelf life of meat. Vacuum packaging can inhibit the growth of spoilage bacteria commonly present on meat, which could affect the meat quality by causing slime formation, off-flavors, and discoloration^4,39^. Among these, psychrotrophic lactic acid bacteria (LAB) are dominant in vacuum-packed meat, and they could affect the quality and shelf life of vacuum-packed meat by producing butyric acid and ethanol, leading to rancid odors and reduced sensory quality^33,40^. This aligns with the sensory rejection of control samples, which had LAB counts exceeding 7 log CFU/g (Table 1). Although curcumin-treated samples were also rejected sensorially, their LAB ranged between 5.55 and 5.90 log CFU/g, which is consistent with the findings of Pennacchia et al.^39^, who found that vacuum-packed beef remained acceptable for longer than the corresponding ones stored in air; however, acceptability did not necessarily associate with low bacterial counts. Furthermore, Borch et al.^33^ mentioned that the correlation between lactic acid bacterial counts and sensorial spoilage is imprecise, suggesting that bacterial levels alone may not reliably predict sensory quality.

**Table 1:** The mean ± SE count (log_10_ CFU/ g) of anaerobic plate counts, lactic acid bacterial counts, and psychrotrophic counts in ground mutton treated with curcumin alone or combined with black pepper during vacuum package storage at 4 °C for 21 days

Microbial Category	Day of storage	Control meatballs	Curcumin-treated meatballs			Black pepper-treated meatballs	Mutton meatballs treated with black pepper + curcumin combination		
			0.3%	0.7%	1%	0.3 %	0.3% + 0.3%	0.3 % + 0.7 %	0.3 % +1 %
Anaerobic plate count (ANPC)	0	6.77^a^ ± 0.16	6.68^a^ ± 0.14	6.60^a^ ± 0.12	6.57^a^ ± 0.16	6.59^a^ ± 0.16	6.55^a^ ± 0.14	6.54^a^ ± 0.12	6.52^a^ ± 0.17
	7	6.91^a^ ± 0.14	6.53^a^ ± 0.12	6.30^a^ ± 0.09	6.28^a^ ± 0.14	6.25^a^ ± 0.14	6.20^a^ ± 0.12	6.04^a^ ± 0.09	5.85^b^ ± 0.14
	14	7.15^a^ ± 0.12	6.06^b^ ± 0.06	5.98^bc^ ± 0.06	5.87^c^ ± 0.12	5.90^c^ ± 0.12	5.70^c^ ± 0.06	5.62^c^ ± 0.06	5.50^c^ ± 0.12
	21	7.39^a^ ± 0.08	5.85^b^ ± 0.03	5.74^b^ ± 0.03	5.50^b^ ± 0.08	5.68^b^ ± 0.08	5.41^b^ ± 0.03	5.28^b^ ± 0.03	5.02^b^ ± 0.09
Lactic Acid bacterial count	0	6.62^a^ ± 0.12	6.57^a^ ± 0.17	6.52^a^ ± 0.16	6.48^a^ ± 0.14	6.51^a^ ± 0.12	6.49^a^ ± 0.17	6.47^a^ ± 0.14	6.46^a^ ± 0.16
	7	6.84^a^ ± 0.09	6.39^a^ ± 0.14	6.33^a^ ± 0.14	6.30^a^ ± 0.12	6.32^a^ ± 0.09	6.28^a^ ± 0.14	6.06^a^ ± 0.12	5.92^b^ ± 0.14
	14	7.02^a^ ± 0.06	6.12^a^ ± 0.12	6.03^b^ ± 0.12	5.91^b^ ± 0.06	5.97^b^ ± 0.06	5.88^b^ ± 0.12	5.76^b^ ± 0.06	5.62^b^ ± 0.12
	21	7.25^a^ ± 0.03	5.90^b^ ± 0.09	5.80^b^ ± 0.08	5.55^b^ ± 0.03	5.71^b^ ± 0.03	5.50^b^ ± 0.09	5.38^b^ ± 0.03	5.20^b^ ± 0.08
Psychrotrophic bacterial count	0	6.71^a^ ± 0.17	6.68^a^ ± 0.12	6.61^a^ ± 0.16	6.58^a^ ± 0.14	6.60^a^ ± 0.16	6.57^a^ ± 0.14	6.54^a^ ± 0.12	6.53^a^ ± 0.16
	7	6.98^a^ ± 0.14	6.40^a^ ± 0.09	6.34^a^ ± 0.14	6.30^a^ ± 0.12	6.32^a^ ± 0.14	6.28^a^ ± 0.12	6.06^a^ ± 0.09	5.89^b^ ± 0.14
	14	7.16^a^ ± 0.12	6.15^b^ ± 0.06	6.04^b^ ± 0.12	5.98^b^ ± 0.06	6.00^b^ ± 0.12	5.83^b^ ± 0.06	5.71^b^ ± 0.06	5.55^c^ ± 0.12
	21	7.42^a^ ± 0.09	5.96^b^ ± 0.03	5.83^b^ ± 0.08	5.50^c^ ± 0.03	5.73^b^ ± 0.08	5.57^c^ ± 0.03	5.36^c^ ± 0.03	5.12^c^ ± 0.08

The shown values denoted the mean of triple measurements ± standard error (SE). Mean values with different superscript letters in the same row are significantly differences at *p* < 0.05.

Previous studies have shown that curcumin and black pepper exhibited potent broad-spectrum antimicrobial activity against Gram-positive and Gram-negative bacteria, by boosting host-mediated immunity and inhibiting bacterial growth via the quorum sensing (QS) system^12,14,16,21,22,41^. Nevertheless, no definite data exist on the mechanism of antibacterial activity of curcumin and black pepper on food spoilage bacteria in vacuum-packed red meat, including anaerobic microorganisms such as lactic acid bacteria and other psychrotrophic species, which may differ according to bacterial type or food matrix. Curcumin’s antibacterial efficacy against food spoilage bacteria, examined in the current study, can be explained by its ability to disrupt bacterial membranes, inhibit the production of virulence factors, and prevent biofilm formation^12^. Black pepper exerts its antibacterial effects by damaging bacterial cell walls and membranes, reducing enzyme activity, and altering bacterial cell morphology^22^. These mechanisms underline the potential of curcumin and black pepper, either alone or in combination, to inhibit the growth of various bacteria, which could significantly improve food safety and prolong the shelf life of food products.

#### Effect of curcumin and black pepper on anaerobic plate count (ANPC)

Microbial growth in vacuum-packaged meat is influenced by environmental factors, including temperature, humidity, and oxygen and carbon dioxide levels. Low vacuum levels in the food industry affect residual oxygen and microbial development. During storage, oxygen levels decrease to about 1%, and carbon dioxide levels rise to 20–25%, inhibiting aerobic bacteria growth and promoting anaerobic and facultative anaerobic bacteria, extending the meat’s shelf life^40^. However, if temperature control is not maintained, pathogenic anaerobic bacteria such as *Clostridium botulinum* can grow^42^, and even an initial count of anaerobic or facultative anaerobic psychrophilic/psychrotrophic bacteria at 10^1^/g or less could proliferate and cause food deterioration^40^. Thus, proper preservation techniques and natural food additives can enhance the safety and shelf life of vacuum-packed meat.

ANPC values for control and treated ground mutton with different concentrations of curcumin, black pepper, or their combination are presented in Table 1. Over a storage period of 21 days, the control meatballs show an increase in ANPC, ranging from 6.77 log_10_ CFU/g on day 0 to 7.39 log_10_ CFU/g on day 21. In contrast, the samples treated with a curcumin-black pepper mixture showed significantly lower ANPC throughout the storage period (Table 1).

On day 0, no significant (*P* > 0.05) differences were observed in ANPC between control and treated samples. However, by day 7, ground mutton treated with 0.3% black pepper + 1% curcumin displayed a substantial decrease in ANPC (*P* < 0.05) by 1.06 logs compared to the control sample (5.85 versus 6.91 log_10_ CFU/g) (Table 1).

By day 14, all treated mutton meatballs with curcumin, black pepper, or their combination revealed a significant decline (*P* < 0.01) in ANPC compared to the control sample. Mutton meatballs treated with a blend of black pepper and curcumin showed the highest reduction in ANPC compared to control and other treated samples. A mixture of 0.3% black pepper + 0.3% curcumin, 0.3% black pepper + 0.7% curcumin, and 0.3% black pepper + 1% curcumin exhibited significant (*P* < 0.05) decline in ANPC by 1.45, 1.53, and 1.65 logs, respectively in comparison to control sample (5.70, 5.62, and 5.50 respectively versus 7.15 log_10_ CFU/g) (Table 1). Likewise, on day 21 of storage, all treated mutton meatballs revealed a significant (*P* < 0.05) reduction in ANPC compared to the control sample, also treated mutton meatballs with 0.3% black pepper + 1% curcumin displayed a substantial decrease in ANPC by 2.37 logs compared to the control sample (5.02 versus 7.39 log_10_ CFU/g) (Table 1).

Overall, the current study revealed that increasing concentrations of curcumin resulted in a progressive decrease in ANPC, indicating its potential as a natural antimicrobial agent when applied in appropriate concentrations. Moreover, the curcumin-black pepper formulation proved to be more effective in reducing ANPC compared to using either additive alone (Table 1).

#### Effect of curcumin and black pepper on lactic acid bacteria (LAB)

Lactic Acid bacteria are facultative anaerobic bacteria that can grow in high CO_2_ concentrations. When aerobic spoilage bacteria are inhibited in vacuum-packed meat, they can become the dominant organism. LAB, such as *Lactobacillus* and *Leuconostoc*, can contribute to chilled raw meat spoilage by producing an undesirable sour aroma and taste^4,5^. Certain LAB strains can adversely affect meat quality during extended storage in vacuum packaging by producing butyric acid, which imparts a rancid or butter-like odor and flavor, as well as ethanol, which can degrade the sensory qualities of the meat and shorten its shelf life^40^. LAB counts for control and treated ground mutton are shown in Table 1. Over a 21-day storage period, the LAB counts in control ground mutton steadily increased from 6.62 to 7.25 log_10_ CFU/g. In contrast, samples treated with curcumin and black pepper significantly reduced LAB count over time (Table 1). On day 0, there were no significant differences (*p* > 0.05) in LAB count between control and treated samples. However, by day 7, ground mutton treated with 0.3% black pepper and 1% curcumin revealed a significant reduction (*P* < 0.05) in LAB count by 0.92 logs compared to the control (5.92 vs. 6.84 log_10_ CFU/g) (Table 1).

By day 14, all treated samples exhibited a significant reduction in LAB counts compared to the control sample, and the combination of 0.3% black pepper + 1% curcumin showed the most significant decrease in LAB counts by 1.40 log compared to the control (5.62 vs. 7.02 log_10_ CFU/g) (Table 1). Similarly, by day 21, mutton meatballs treated with a black pepper-curcumin blend revealed a substantial reduction in LAB counts compared to control and other treated samples. On day 21, a formulation of 0.3% black pepper + 0.3% curcumin, 0.3% black pepper + 0.7% curcumin, and 0.3% black pepper + 1% curcumin revealed a significant (*P* < 0.01) reduction in LAB counts by1.75, 1.87, and 2.05 logs, respectively compared to the control sample (5.50, 5.38, and 5.20 respectively versus 7.25 log_10_ CFU/g) (Table 1).

The current findings demonstrated that the curcumin-black pepper blend effectively inhibited LAB growth, thereby improving safety and prolonging the shelf life of vacuum-packed meat. These findings contrast with earlier research, where essential oil from Curcuma longa (turmeric) was reported to be less effective in inhibiting LAB in vacuum-packed, cooked sausage stored at 4 °C compared to other plant essential oils^43^. Similarly, another study reported that meatballs treated with 4% turmeric exhibited higher LAB counts than the control^44^. These discrepancies highlight the variability in antimicrobial efficacy depending on the food matrix, storage conditions, and the concentrations of curcumin used.

#### Effect of curcumin and black pepper on psychrotrophic bacteria

Psychotropic bacteria are the main microorganisms accounting for meat spoilage during refrigerated storage. In vacuum-packaged meat, psychrotrophic bacteria, like *Enterobacteriaceae*, *Brochothrix thermosphacta*, and *Shewanella putrefaciens*, could proliferate and cause food spoilage^40^. The mean values (log_10_ CFU/g) of psychrotrophic bacterial counts for control and treated ground mutton with different concentrations of curcumin, black pepper, or their combination are displayed in Table 1. The mean values of psychrotrophic bacteria counts of control (untreated) ground mutton increased during storage from 6.71 to 7.42 log_10_ CFU/g, while the mean values of treated meatballs decreased significantly (*P* < 0.01) during the 21-day storage (Table 1). On day 0, there were no significant differences in psychrotrophic bacterial count between control and treated samples. Nevertheless, by day 7, ground mutton treated with 0.3% black pepper and 1% curcumin showed a significant decline in the psychrotrophic count by 1.09 logs compared to the control (5.89 vs. 6.98 log_10_ CFU/g) (Table 1).

On day 14, all treated samples exhibited a significant reduction in psychrotrophic counts compared to the control, and a formulation of 0.3% black pepper and 1% curcumin revealed a substantial (*P* < 0.01) reduction in psychrotrophic counts by 1.61 logs compared to the control sample (5.55 vs. 7.16 log_10_ CFU/g) (Table 1). Similarly, on day 21, mutton meatballs treated with a mixture of black pepper and curcumin displayed a significant reduction in psychrotrophic counts compared to the control and other treated meatballs; specifically, a mixture of 0.3% black pepper + 0.3% curcumin, 0.3% black pepper + 0.7% curcumin, and 0.3% black pepper + 1% curcumin showed a significant (*P* < 0.01) reduction in psychrotrophic counts by 1.85, 2.06, and 2.3 logs, respectively compared to the control sample (5.57, 5.36, and 5.12 respectively versus 7.42 log_10_ CFU/g) (Table 1).

In this context, it has been reported that the decimal reduction of total aerobic psychrotrophic bacteria in the chicken breast fillets after 16 days of storage at 4 °C was 2.6 for vacuum packaging and edible coatings containing 2% black pepper seeds and 3.9 for vacuum packaging and edible coatings containing 2% turmeric extract^45^. The same study also mentioned that the edible coating containing turmeric extract was more effective than those containing black pepper extract in reducing psychotropic counts and extending the shelf life of chicken breasts^45^. Likewise, the study of Qian et al. found that curcumin and piperine combined with vacuum packaging could effectively suppress the growth of psychotropic bacteria in salmon during cold storage^41^.

### Antimicrobial effect of curcumin and black pepper against foodborne pathogens inoculated into ground mutton

Curcumin is a natural antimicrobial agent with strain-specific activity that varies depending on the bacterial species and their structural characteristics^14^. Curcumin’s antimicrobial activity may be related to its ability to disrupt bacterial quorum-sensing systems, inhibit biofilm formation, prevent bacterial adhesion to host receptors in several species, and enhance the membrane permeabilization of bacteria^12^. Additionally, curcumin could inhibit DNA replication, alter gene expression, and interfere with cell division by disrupting FtsZ protofilament polymerization and GTPase activity in various species, including *E*. *coli* and *S*. *aureus*^14^. These mechanisms are effective against a broad range of bacteria.

Our findings revealed significantly higher activity of curcumin against Gram-positive (*S*. *aureus*) than Gram-negative bacteria (*S*. Typhimurium and *E*. *coli* O157:H7). Specifically, treatments with 1% curcumin, 0.3% black pepper, and curcumin-black pepper blends completely inhibited *S*. *aureus* growth by day 21 of storage (Fig. 5A and B). Similarly, Tyagi et al. found that 100 μM curcumin caused 100% killing of *S*. *aureus* after 2 h exposure by perturbing the bacterial membrane integrity^46^. Curcumin and black pepper exhibit stronger antibacterial activity against Gram-positive bacteria than Gram-negative bacteria due to structural differences in their cell walls. Gram-positive bacteria possess a thick peptidoglycan layer but lack an outer membrane, facilitating penetration, while Gram-negative bacteria have an outer membrane that acts as a permeability barrier, reducing compound effectiveness^14^. On the other hand, Tyagi et al. demonstrated that curcumin could induce membrane permeabilization in *E*. *coli* and *S*. *aureus*, despite their significantly different cell wall structures^46^.

**Fig. 3:**
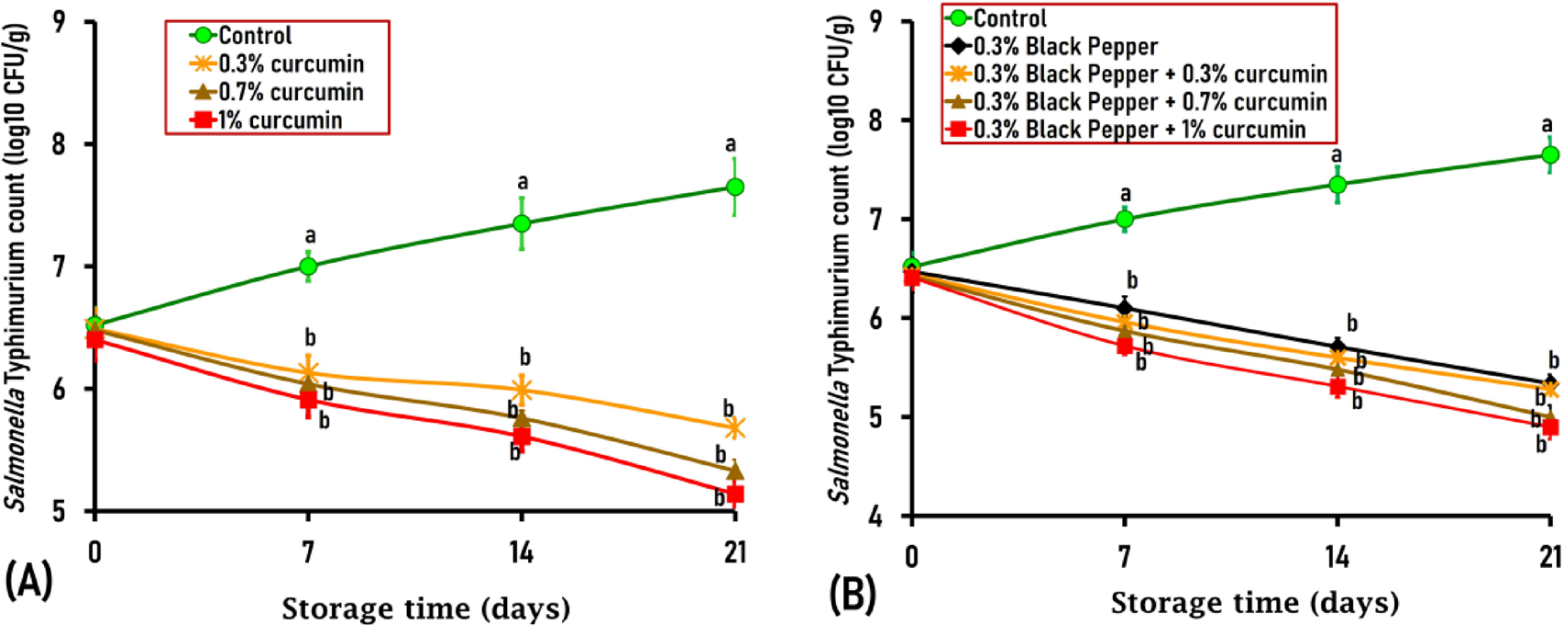
Antimicrobial effect of curcumin at different concentrations of 0.3%, 0.7%, and 1% (**A**), black pepper (0.3%), and the combination of black pepper and curcumin (**B**) on *Salmonella enterica* serovar Typhimurium, artificially inoculated into ground mutton stored under vacuum-packed conditions at 4 °C for 21 days. The standard error (SE) is represented by Vertical bars; Mean values with different letters among the various treated groups are significantly different at *P* < 0.05 or *P* < 0.01.

**Fig. 4:**
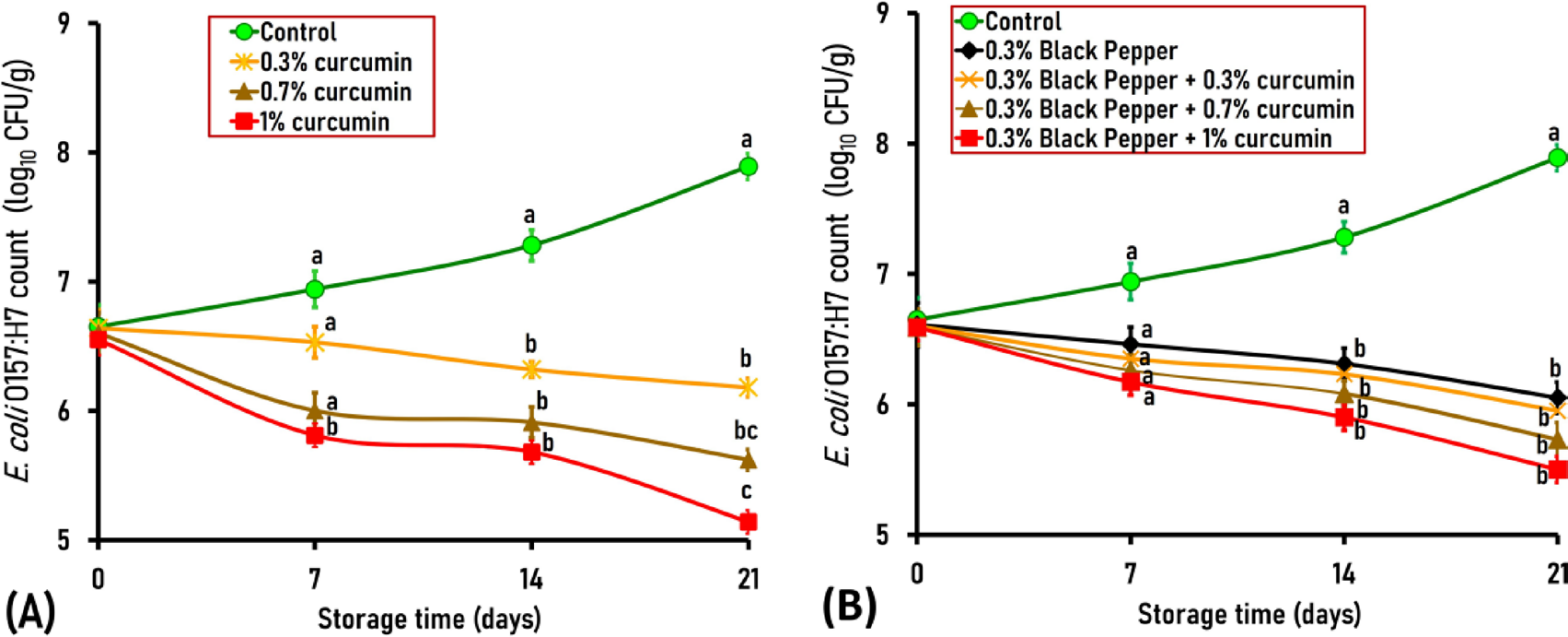
Antimicrobial effect of curcumin at different concentrations of 0.3%, 0.7%, and 1% (**A**), black pepper (0.3%), and the combination of black pepper and curcumin (**B**) on *Escherichia coli* O157:H7, artificially inoculated into ground mutton stored under vacuum-packed conditions at 4 °C for 21 days. The standard error (SE) is represented by vertical bars; Mean values with different letters among the various treated groups are significantly different at *P* < 0.05 or *P* < 0.01.

By day 21 of storage, mutton samples treated with 0.3% black pepper + 1% curcumin showed significant reductions of 2.75 and 2.39 log CFU/g in *S*. Typhimurium and *E*. *coli* O157 by 2.75 and 2.39 logs, respectively, compared to the control sample (Figs. 3 and 4). Achieving > 2 log reductions in foodborne pathogens is microbiologically significant because it could induce complete inhibition of pathogens, especially when combined with other preservation methods. Furthermore, it has been suggested that a higher concentration of curcumin or its prolonged exposure may be essential for complete bacterial elimination^46^. Further research is needed to ascertain whether sub-lethal exposure to curcumin and black pepper enables surviving bacterial cells to activate stress responses or develop resistance, potentially resulting in rebound growth during storage.

#### Effect of curcumin and black pepper against Salmonella Typhimurium

On the first day of the experiment (day 0), the initial count of *Salmonella* Typhimurium inoculated in control and herbal-treated ground mutton ranged from 6.52 to 6.40 log_10_ CFU/g, with no significant differences observed between the control and treated samples (Fig. 3A and B). However, by day 7, treated mutton meatballs with curcumin, black pepper, or their combination exhibited a significant decline in *Salmonella* counts compared to the control sample. A formulation of 0.3% black pepper + 0.3% curcumin, 0.3% black pepper + 0.7% curcumin, and 0.3% black pepper + 1% curcumin showed a significant (*P* < 0.05) reduction in *Salmonella* counts by 1.04, 1.13, and 1.28 logs, respectively compared to the control sample (5.96, 5.87, and 5.72 respectively versus 7.0 log_10_ CFU/g) (Fig. 3B).

On day 14, all treated samples revealed significant decreases in *Salmonella* counts compared to the control sample and a combination of 0.3% black pepper + 0.3% curcumin, 0.3% black pepper + 0.7% curcumin, and 0.3% black pepper + 1% curcumin exhibited a substantial (*P* < 0.01) reduction in *Salmonella* counts by 1.75, 1.87, and 2.04 logs, respectively compared to the control sample (5.60, 5.48, and 5.31 respectively versus 7.35 log_10_ CFU/g) (Fig. 3B). Similarly, by day 21, mutton meatballs treated with a black pepper-curcumin blend displayed a significant decrease in *Salmonella* counts compared to the control and other treated meatballs. A mixture of 0.3% black pepper + 0.3% curcumin, 0.3% black pepper + 0.7% curcumin, and 0.3% black pepper + 1% curcumin showed a significant (*P* < 0.01) reduction in *Salmonella* counts by 2.37, 2.65, and 2.75 logs, respectively compared to the control sample (5.28, 5.0, and 4.9 respectively versus 7.65 log_10_ CFU/g) (Fig. 3B).

Similar to our findings, a study in Turkey indicated that adding curcumin at concentrations of 0.5%, 1%, and 2% had lowered *S*. Typhimurium counts in minced meat at the end of 7 days of storage by approximately 1.99, 3.18, and 3.28 log respectively compared to control (untreated) sample (3.72, 2.53, and 2.43 respectively versus 5.71 log_10_ CFU/g)^24^. Likewise, another study in Turkey revealed that adding 1%, 2%, and 3% curcumin to chicken meat samples reduced the *S*. Typhimurium by 2.37, 2.71, and 2.84 log_10_ CFU/g, respectively, by the sixth day of storage^23^. The current findings highlighted the potential effect of curcumin and black pepper as promising natural antimicrobial agents for controlling *Salmonella* growth in the meat industry.

#### Effect of curcumin and black pepper against E. coli O157:H7

The initial count of inoculated *E*. *coli* O157:H7 in control and treated ground mutton ranged from 6.65 to 6.55 log_10_ CFU/g on day 0 of the experiment (Fig. 4A and B). No significant differences in *E*. *coli* O157:H7 counts were detected between the control and treated meatballs, neither on day 0 nor on day 7 of storage (Fig. 4A and B), except for 1%-curcumin-treated samples on day 7 at which exhibited 1.13 log lower than the control. Nevertheless, by day 14, mutton meatballs treated with curcumin, black pepper, or their combination revealed a significant reduction in *E*. *coli* O157:H7 counts compared to the control. A blend of 0.3% black pepper + 0.3% curcumin, 0.3% black pepper + 0.7% curcumin, and 0.3% black pepper + 1% curcumin displayed a significant (*P* < 0.05) reduction in *E*. *coli* O157:H7 counts by 1.05, 1.20, and 1.38 logs, respectively compared to the control sample (6.23, 6.08, and 5.90 respectively versus 7.28 log_10_ CFU/g) (Fig. 4B).

By day 21 of storage, all treated mutton meatballs exhibited a substantial reduction in *E*. *coli* O157:H7 counts compared to the control sample, specifically, ground mutton treated with a blend of black pepper and curcumin revealed the most significant decrease in *E*. *coli* O157:H7 counts compared to the control and other treated meatballs. A combination of 0.3% black pepper + 0.3% curcumin, 0.3% black pepper + 0.7% curcumin, and 0.3% black pepper + 1% curcumin displayed a significant (*P* < 0.01) reduction in *E*. *coli* O157:H7 counts by 1.94, 2.16, and 2.39 logs, respectively compared to the control sample (5.95, 5.73, and 5.50 respectively versus 7.89 log_10_ CFU/g) (Fig. 4B).

In this context, Sandıkçı Altunatmaz et al. mentioned that the addition of 0.5%, 1% and 2% curcumin to minced meat had lowered *E*. *coli* O157:H7 counts at the end of seven days by about 1.07, 2.47, and 2.59 log respectively compared to the control sample (3.78, 2.38, and 2.26 respectively versus 4.85 log_10_ CFU/g)^24^. Likewise, Zhang et al. demonstrated that black pepper essential oil (BPEO) possessed potent antimicrobial activity against meat-borne *E*. *coli*^37^. The same researchers also observed that a treatment at 1 × MIC (with the minimum inhibitory concentration of BPEO being 1.0 μL/mL) led to a significant 16.20% reduction in *E*. *coli* counts (from 6.11 to 5.12 log CFU/mL) after 12 h. Those findings underscore the potent antimicrobial efficacy of curcumin and black pepper as natural food preservatives against *E*. *coli* O157:H7.

#### Effect of curcumin and black pepper against methicillin-resistant Staphylococcus aureus (MRSA)

**Fig. 5:**
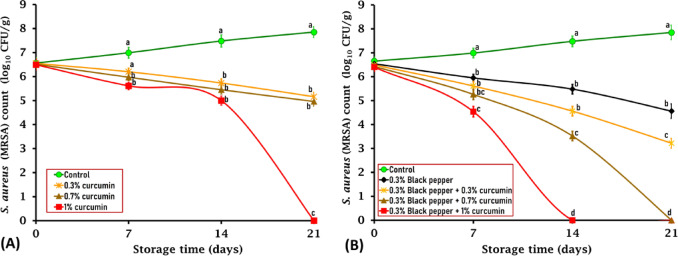
Antimicrobial effect of curcumin at different concentrations of 0.3%, 0.7%, and 1% (**A**), black pepper (0.3%), and the combination of black pepper and curcumin (**B**) on methicillin-resistant *S. aureus* (MRSA), artificially inoculated into ground mutton stored under vacuum-packed conditions at 4 °C for 21 days. The standard error (SE) is represented by vertical bars; Mean values with different letters among the various treated groups are significantly different at *P* < 0.05 or *P* < 0.01.

On day 0 of the experiment, there was no significant difference in the initial counts of inoculated *S*. *aureus* between the control and treated ground mutton, which ranged from 6.56 to 6.40 log_10_ CFU/g (Fig. 5A and B). However, by day 7, mutton meatballs treated with 0.7% curcumin, 1% curcumin, 0.3% black pepper, combinations of 0.3% black pepper + 0.3% curcumin, 0.3% black pepper + 0.7% curcumin, and 0.3% black pepper + 1% curcumin showed significant (*P* < 0.05) reductions in *S*. *aureus* counts by 1.02, 1.38, 1.04, 1.38, 1.63, and 2.45 logs, respectively compared to the control sample (5.97, 5.61, 5.95, 5.61, 5.26, and 4.54 respectively versus 6.99 log_10_ CFU/g) (Fig. 5A and B).

By day 14, the combination of 0.3% black pepper and 1% curcumin completely inhibited the growth of *S*. *aureus* (0 counts) (Fig. 5B). Other treatments, including curcumin at concentrations of 0.3%, 0.7%, and 1%, as well as black pepper either alone at a concentration of 0.3% or in combination with curcumin at concentrations of 3% or 0.7%, displayed a significant (*P* < 0.01) decline in *S*. *aureus* counts by 1.75, 2.03, 2.48, 2.0, 2.92, and 3.96 logs, respectively compared to the control sample (5.73, 5.45, 5.00, 5.48, 4.56, and 3.52 respectively versus 7.48 log_10_ CFU/g (Fig. 5A and B).

By day 21, ground mutton samples treated with 1% curcumin powder, and those treated with a combination of 0.3% black pepper and 0.7% curcumin revealed complete inhibition (0 counts) of *S*. *aureus* growth, while ground mutton treated with 0.3% curcumin, 0.7% curcumin, 0.3% black pepper alone and a mixture of 0.3% black pepper and 0.3% curcumin showed a significant (*P* < 0.01) decline in *S*. *aureus* counts by 2.69, 2.89, 3.29, and 4.63 logs, respectively compared to the control sample (5.16, 4.96, 4.56, and 3.22 respectively versus 7.85 log_10_ CFU/g) (Fig. 5A and B).

Consistent with our results, Sandıkçı Altunatmaz et al. found that addition of 0.5%, 1%, and 2% curcumin displayed a significant (*P* < 0.05) decline in *S*. *aureus* counts in minced meat after 7 days of storage by 2.89, 2.84, and 4.15 log respectively compared to control (untreated) sample (2.62, 2.67, and 1.36 respectively versus 5.51 log_10_ CFU/g)^24^. Moreover, Teow et al. revealed that curcumin possesses significant antimicrobial effects against methicillin-resistant *Staphylococcus aureus* (MRSA) and methicillin-sensitive *Staphylococcus aureus* (MSSA) via perturbing the bacterial membrane integrity^47^. Black pepper could affect membrane permeability and integrity, leading to bacterial cell wall lysis, membrane expansion and destabilization, separation of the cell membrane from the cell wall, and subsequent loss of intracellular materials, resulting in leakage and cell death^37^. Therefore, the current study elucidated the efficacy of using curcumin and black pepper as natural antimicrobial agents against *S*. *aureus*.

## Conclusion

The current research is one of the few studies investigating the efficacy of curcumin, black pepper, or their combination on the sensory attributes and microbiological quality of vacuum-packed meat. The current findings concluded that mutton meatballs treated with black pepper alone or black pepper and curcumin together were more sensorially acceptable than meatballs treated with curcumin alone, while using 1% curcumin alone had the least flavor score. The combination of 0.3% black pepper and 1% curcumin showed the highest antimicrobial effect against the artificially inoculated foodborne pathogens as well as food spoilage bacteria. Consequently, because of the potent antimicrobial characteristics of curcumin and black pepper, along with their synergistic impact which improves their joint capacity to combat foodborne pathogens and slow down spoilage bacteria they could be used as promising natural additives position them as suitable options for commercial or industrial use in the food industry, aligning closely with consumer demand, to enhance the sensory quality, safeguarding food safety, and extend the shelf life of vacuum-packaged stored meat products. Further studies are required about the mechanism of action of curcumin against foodborne pathogens and its application in the food industry, including consumer acceptance and regulatory approval.

## Electronic supplementary material

Below is the link to the electronic supplementary material.

Supplementary Material 1

## Data Availability

All data supporting the findings of this study are included within the article and its supplementary information files. No datasets were generated or analyzed during the current study. Any additional information is available from the corresponding author upon reasonable request.
