# Supplementary material for: Antimicrobial effect of curcumin, alone or in combination with black pepper, against foodborne pathogens in vacuum-packed ground mutton
Source: Sci Rep. 2025 Jul 8;15:24373. doi: 10.1038/s41598-025-08350-2 (PMC12238417; doi:10.1038/s41598-025-08350-2)
Supplement: Supplementary file 1 — Supplementary Material 1 [file 41598_2025_8350_MOESM1_ESM.docx]

**Highlights**

- Curcumin and black pepper enhanced the sensory quality of vacuum-packed ground mutton
- Curcumin alone or with black pepper delayed the bacterial spoilage of ground mutton
- Curcumin alone or with black pepper improved the shelf life of ground mutton
- Curcumin with black pepper reduced *S*. Typhimurium and *E*. *coli* by > 2 log in ground mutton
- Curcumin alone or with black pepper fully inhibits *S*. *aureus* growth in ground mutton
